# Lavender and Black Pine Waste as Additives Enhancing Selected Mechanical and Hygrothermal Properties of Cement Mortars

**DOI:** 10.3390/ma17225475

**Published:** 2024-11-09

**Authors:** Jarosław Strzałkowski, Petrini Kampragkou, Maria Stefanidou, Agata Markowska-Szczupak, Elżbieta Horszczaruk, Anna Głowacka

**Affiliations:** 1 Faculty of Civil and Environmental Engineering, West Pomeranian University of Technology in Szczecin, Piastów Ave. 50A, 70-311 Szczecin, Poland; elzbieta.horszczaruk@zut.edu.pl (E.H.); anna.glowacka@zut.edu.pl (A.G.); 2 Laboratory of Building Materials, Department of Civil Engineering, Aristotle University of Thessaloniki, P.O. Box 456, Gr-54124 Thessaloniki, Greece; petrinik@civil.auth.gr (P.K.); stefan@civil.auth.gr (M.S.); 3 Faculty of Chemical Technology and Engineering, West Pomeranian University of Technology in Szczecin, Piastów Ave. 42, 71-065 Szczecin, Poland; agata.markowska@zut.edu.pl

**Keywords:** cement mortars, bio-powders, lavender powder, black pine powder, hydrothermal properties of mortars, mechanical properties of mortars

## Abstract

The paper presents the mechanical and hygrothermal properties of cement mortars containing bio-powders made from lavender waste and black pine wood. The wastes were mechanically ground with a hammer mill to a fraction not exceeding 0.5 mm and then dried in air-dry conditions. The influence of bio-additives in amounts of 1.5% and 2.5% of the overall mortar volume was tested. The aim of the paper was to determine the impact of bio-additives on the mechanical and hygrothermal properties of the tested cement mortars. This publication included tests of compressive and flexural strength, elastic modulus, water absorption, absorption due to capillary rise, sorption and desorption properties, thermal properties, microstructural tests using mercury intrusion porosimetry and SEM, and EDS. The main conclusions of the research indicate that mortars with both 1.5% and 2.5% bio-powders are characterized by strong bactericidal properties, lower sorption properties at high air humidity, lower thermal conductivity, reduced compressive strength by 22–27%, no significant effect on the flexural strength, and significant reduction in capillary action of mortars both with short-term and long-term water exposure.

## 1. Introduction

Natural-origin post-production waste presents a significant challenge in terms of disposal and greenhouse gas emissions, including carbon dioxide. A potential solution to mitigate this issue involves utilizing such waste as raw materials for manufacturing building materials.

The literature review [1] provided an extensive overview of biowaste applications, drawing important conclusions regarding the viability of agro-industrial waste in construction. Findings indicate that insulation materials made from agro-industrial waste show considerable potential as alternatives to conventional insulators, like polystyrene or mineral wool. The analysis covers various biowaste types, including husks, straw, sawdust, and wood flour, which can be processed into insulation boards with effective thermal properties.

A notable advantage of these materials lies in their sustainability, as they enable the repurposing of agro-industrial waste, thereby lessening environmental impact. These materials are also characterized by favorable thermal insulation and humidity control properties, contributing to enhanced building energy efficiency. Nonetheless, the review [1] also addressed the challenges impeding the broad adoption of such materials in European markets, particularly related to technological constraints and compliance with standards and regulations for new insulation materials. Further information on plant-fiber-based materials and their properties can be found in sources [2,3].

The review [4] examined the impact of incorporating biowaste fibers into 3D-printed concrete. Integrating plant fibers in printed concrete can enhance its flexural strength and crack resistance, while also potentially benefiting the rheological properties of the concrete mixture—an essential factor in the 3D printing process. However, the review also highlighted challenges related to the 3D printing of recycled concrete that includes plant fibers, particularly in controlling the printing process to achieve consistent quality and in optimizing the concrete mix parameters to account for the addition of plant fibers.

Another notable study [5] investigated how natural materials can inspire the design of advanced fiber-reinforced composites, detailing advances in 3D printing and other fabrication methods that enable precise control over fiber orientation and matrix composition—factors critical to replicating natural toughening mechanisms.

Further progress in the use of natural fibers in sound-absorbing materials is covered in review [6]. Such materials show promise as replacements for traditional sound-absorbing products, like acoustic foam and mineral fibers, largely due to their porous structure, which facilitates sound wave absorption.

The influence of fibers in plasters and renders is comprehensively analyzed in review [7]. The addition of plant fibers has been shown to enhance the tensile strength and crack resistance of mortars; however, the review underscores the importance of the optimal selection of mixture components to achieve targeted properties. Environmental conditions and locally available raw materials also play a crucial role in designing earth mortars. Further studies on the impact of biofiber additives on plaster properties are presented in [8], where findings indicated that incorporating A. dealbata in gypsum plaster significantly increases hygroscopicity. In some cases, the moisture buffering capacity of the reference mortar triples, showing comparable performance with highly hygroscopic clay-based plasters.

In the group of bio-reinforced cement mortars, the inclusion of wood fibers (from black pine and beech species) is noted to enhance both volume stability and thermal performance [9]. Additionally, fibers from jute, kelp, and coconut improved fracture energy and flexural strength [10]. Research presented in [11] explores the utilization of mild hydrothermally treated wood fibers (black pine and beech) as reinforcements for cement mortars, assessing long-term behavior under varied aging conditions. Results indicate the satisfactory performance of biofibers in alkaline environments, maintaining specimen durability across multiple hardening scenarios (wet, freeze-thaw cycles, and outdoor exposure). These reinforced mortars exhibit improved volume stability, flexural strength, capillary absorption (notably after 365 days with black pine additives), thermal properties, and breathability.

Further, studies in [12] investigated the fatigue performance of fiber-reinforced geopolymer concrete (GPC), emphasizing its potential as an alternative to traditional Portland cement concrete for structural applications.

In publication [13], the results of substituting synthetic fibers with natural fibers in geopolymer mortar reinforcement are presented. The study found that natural fibers, such as those from pine cones, can serve as effective replacements for glass fibers, enhancing the mechanical properties and crack resistance of geopolymer mortars. This research also underscores the ecological advantages of natural fibers, which are biodegradable and exhibit a lower environmental impact than synthetic alternatives. Furthermore, natural fiber use has potential cost-saving and waste-reduction benefits in production. Mechanical properties of concrete incorporating other biowaste materials, such as corncob ash or crab shell powder, are explored in the publication [14].

Another study [15] examined the use of natural fibers in stabilizing soil blocks for sustainable construction applications. Areca, coir, and flax fibers were chosen to reinforce the soil blocks, with 2.5% cement employed for stabilization. The researchers investigated the mechanical strength and durability of soil blocks, aiming to promote eco-friendly building solutions by enhancing soil blocks’ strength, water resistance, and overall sustainability.

Another significant aspect of utilizing biowastes concerns their environmental impact. Publication [16] investigated the potential of biowaste materials in construction applications, specifically focusing on their thermal properties. It examined how agricultural and industrial waste can be repurposed for building insulation and other applications. Using Life Cycle Assessment (LCA), the study assessed the sustainability and environmental benefits of biowaste materials, noting their reduced carbon footprint and enhanced energy efficiency compared to conventional materials.

Hemp is among the most widely studied bio-additive for concrete and mortar. Review [17] provided a comprehensive overview of recent advancements in hemp concrete research, also addressing challenges such as variability in fiber quality and the need for standardized processing methods to ensure consistent performance. Integrating hemp fibers shows promise in advancing the sustainability and performance of concrete in construction. Additional insights into hemp concrete are available in [18,19,20].

Coconut fibers, or coir, are also popular in mortar and concrete reinforcement. Study [21] evaluated the potential of coir as a reinforcement material, highlighting its advantageous properties, such as high tensile strength, durability, and environmental friendliness. The review discussed coir’s contributions to enhancing crack resistance, reducing shrinkage, and improving the mechanical properties of cement composites, while also addressing challenges, including fiber treatment, bonding with the cement matrix, and long-term durability.

Straw fibers are among the most frequently used biofibers in Mediterranean countries [22]. A recent study explored incorporating straw fibers into adobe bricks, examining how their addition impacts hygrothermal properties, such as moisture regulation and thermal insulation, alongside mechanical strength. The findings indicate that straw fiber reinforcement enhances the energy efficiency and structural integrity of adobe bricks, making them viable for sustainable construction in semi-arid climates. Review [23] provides an in-depth analysis of the thermal and mechanical behavior of straw-based construction materials, but it also highlights the need for further research on sound resistance, energy performance, cost implications, and the mechanisms of interior air moisture regulation in straw-based buildings.

Lavender fibers, another post-production waste from the cosmetics industry, also show potential for application in mortars and concretes. Publication [24] investigated the use of distilled lavender stalks—an essential oil production byproduct—as bioaggregates in sustainable construction materials. The study evaluated the hygrothermal properties, mechanical performance, and chemical interactions of lavender stalks with a mineral pozzolanic binder. Further, the potential use of lavender fibers as reinforcement in lime-based mortars is explored in [25]. This research compares two incorporation techniques (fibers dispersed within the mortar or arranged as a fiber net between two mortar layers), with experimental results indicating positive effects on the physical, mechanical, and thermal properties of the reinforced mortars. Additional research explored the integration of lavender straws in earth-based building materials. When used as bioaggregates in earth bricks, lavender straws offer a sustainable alternative to traditional building materials by enhancing thermal and hygrothermal performance. Should the mechanical properties and durability of these bioaggregate earth bricks meet building standards, they could become a prominent choice in sustainable construction practices.

Studies [26,27] examined the effects of incorporating pine needle fibers into concrete and its resulting mechanical performance. Findings indicate that pine needle fibers can improve compressive strength, splitting strength, and modulus of rupture, while also enhancing ductility and toughness, suggesting the viability of pine needle fibers in creating a novel plant-fiber-reinforced concrete composite. Additional testing of red pine needle fibers in self-compacting ultra-high-performance concrete (UHPC) [28] suggested they can effectively improve strength and crack resistance without compromising the material’s self-compacting characteristics. The elemental composition of pine needles is thoroughly analyzed in [29].

Research has also examined the use of pine ash [30] and pine resin [31] in concrete. These studies reveal that pine ash can significantly enhance strength, workability, and durability, while pine resin increases flexural strength and crack resistance. Together, these additives contribute to concrete with improved insulation properties and enhanced thermal stability. Other studies on the use of pine waste are presented in [32]. The authors employed artificial neural networks (ANNs) to predict the strength outcomes of the composite material, using various input parameters such as fiber content, soil type, and construction demolition waste proportions. The findings suggest that the addition of pine needle fibers significantly improves the mechanical properties of the soil.

In summary, the literature review revealed that only a limited number of studies have investigated the incorporation of lavender biowaste powder and black pine powder into concrete. There is a lack of results regarding the impact of lavender and black pine powder on the moisture properties of such prepared mortars. Additionally, studies on the bactericidal properties of these types of additives are lacking. These are waste materials that producers in Greece face challenges with. Their use in the construction industry could bring benefits not only through waste reduction but also by improving selected properties of such prepared mortars.

This paper aims to address a significant research gap by presenting a comprehensive analysis of the mechanical, thermal, and moisture-related properties of cement-based materials containing biowastes. Specifically, this study focuses on water absorption, capillary rise, and sorption and desorption characteristics—areas that are rarely examined in research on cementitious materials with biowaste additives, particularly lavender and black pine wood.

To this end, a series of cement mortars containing various bio-powders was evaluated. The key research questions addressed in this study are as follows:

- How does increasing the content of bio-powders in mortar influence its physical properties, such as water absorption, capillary rise, sorption properties, and thermal behavior?
- What is the relationship between the presence of bio-materials and the mechanical properties of cement mortars?
- How do the analyzed waste materials affect the bactericidal properties of the mortar?
- What correlations exist between microstructural parameters and moisture-related properties?

This paper seeks to answer these questions, along with additional inquiries regarding cement mortars containing different concentrations of lavender and black pine powders.

In summary, this study aims to fill the research gap concerning mortars incorporating these specific biowastes in powdered form. The results presented in this publication provide insights into whether such mortars exhibit sufficiently good mechanical properties and assess the impact of biowaste on the moisture-related characteristics of these materials. In addition, this study evaluates the resistance of these materials to the growth of microorganisms. The advantage of the solution presented in this publication, which involves the use of biowaste in mortars, lies in two key aspects: on one hand, it reduces the amount of waste by incorporating it into the mortar itself, and on the other, it has a beneficial impact on selected properties of the entire composite.

## 2. Materials and Methods

The research program involved the preparation of five mortar mixtures using CEM I 42.5R cement, silica sand with a grain size of up to 4 mm, and tap water. The first composite, designated as the reference mix (REF), had a cement-to-sand ratio of 1:2.5 by weight. All the mixes had a w/c ratio equal to 0.45. The aforementioned design was formulated based on the insights gained from the analysis of various types of mortars and prior research findings [9,11,25,33].

The aggregate utilized in the tests consisted of ordinary river silica sand, which was dried in an oven. Subsequently, the entire material was sieved to retain only the fraction with a maximum particle size of 4 mm, and any contaminants that could potentially impact the reliability of the test results were eliminated.

In subsequent composites, the volume of biowastes was proportionally increased, with a simultaneous and proportional increase in superplasticizer to maintain similar workability of all the mixes, keeping the w/c ratio stable.

The composition of individual mixtures is shown in Table 1. The plasticizer used in the prepared mixtures was an aqueous solution of polycarboxylate polymer. The recommended dosage was 0.3 to 2.0% in relation to the cement mass. It was necessary to use a high dosage of superplasticizer to ensure the proper workability of specimens with lavender, which significantly impacted the individual properties of this mixture.

The bio-additives used in this research were a fine fraction of particles prepared from post-production waste of lavender (L) and black pine wood (BP). The waste was mechanically ground with a hammer mill to a fraction not exceeding 0.5 mm and then dried in air-dry conditions (climatic chamber with stable conditions: 20 ± 2 °C and 60 ± 5% relative humidity) for about 60 days until the achievement of a constant mass. The lavender bio-material (*Lavandula* sp.) was obtained as a steam distillation process waste of the essential oils production in North Greece. The black pine wood material (*Pinus nigra* L.) was derived as a wood processing industry residue from Central Greece (Kalampaka region). The chemical composition of the biowastes is presented in Table 2.

Table 2 lists the elements present in both types of powders. Apart from carbon and oxygen, which constitute over 98% of the elemental composition of both materials, a noticeable presence of Ca and K in lavender was recorded. Black pine turned out to be much purer due to its elemental composition. In the case of lavender, the composition includes, among others, zinc, which has strong antibacterial properties. Similarly, the concentrations of Cu and Mg present in both powders may also potentially affect the antimicrobial properties of the mortar additive.

### 2.1. Flow Table Test

A flow table workability test followed the EN 1015-3 standard [34]. The flow value was determined by measuring the mean diameter of a test specimen of fresh mortar placed on a designated flow table disc. The specimen underwent 15 vertical impacts by elevating the flow table and allowing the sample to fall freely from a specified height. The diameter of the mortar spread was measured in two perpendicular directions to ensure accuracy. Following the completion of the first measurement, all procedures were repeated to obtain a second measurement. The final results, expressed in millimeters with a precision of up to one millimeter, represented the averaged values of the two measurements [34].

### 2.2. Specimen Preparation and Curing

Following the aforementioned tests, the prepared mortar mixtures were placed into molds in two layers, with each layer being mechanically compacted sequentially. Prism-shaped 40 × 40 × 160 mm and plate-shaped 200 × 200 × 25 mm molds were compacted using a vibrating table. The specimens were demolded after 24 h and stored in a climatic chamber at a temperature of approximately ~20 °C, with humidity exceeding 95%, for 28 days of curing. Then, until 90 days, all the specimens were placed in a climatic chamber with a temperature of ~20 °C and relative humidity 50 ± 5%. For each composition, 15 prisms and one plate specimen were prepared.

### 2.3. Physical Properties (Density, Absorption, Open Porosity and Apparent Specific Gravity)

The volume density test followed the EN 1015-6 [35] standard on 40 × 40 × 160 mm prism specimens. Measurements were conducted on three specimens of each type of composite, evaluated in three distinct moisture states:

- Dried—after drying to a constant mass at a temperature of 70 °C.
- Water-saturated—after 48 h of immersing the specimens in distilled water.
- Air-dry—after two months of conditioning the specimens in air-dry conditions (20 °C and RH < 50%).

Furthermore, the specimens’ absorption, open porosity, and apparent specific values were determined following RILEM CPC 11.3 [36]. More specifically, after 28 and 90 hardening days, the under-vacuum liquid absorption method was implemented for small specimens (half prisms), including drying the samples, removing the air from the samples’ pores (with vacuuming), and covering them with water. Through the recording of their weight changes (due to the penetration of water inside the open pores) and considering Archimedes’ principle, the specimens’ absorption, open porosity, and apparent specific characteristics were measured [37,38].

### 2.4. Thermal Properties Tests

The thermal properties test was performed using two methods. For obtaining high accuracy of thermal conductivity λ results, a heat flow meter apparatus by EN 12667 [39] (Thermtest-HFM 100 Series device, Veddige by 2, 432 68 Veddige, Sweden https://thermtest.com/) was applied to the plate specimens at the age of 28 and 90 days. Analytically, the λ values were recorded at 10 °C and 20 °C (mean heating temperature), applying a difference of 10 °C to the instrument’s heating-cooling plates each time. Before executing the λ measurements, the plates were placed in a climatic chamber with stable conditions (temperature ~20 °C and relative humidity 50 ± 5%) until the achievement of constant mass (the mass changes were recorded with successive weight measurements). Then, their surfaces were sanded to achieve smooth and parallel sides. This drying-smoothing process aimed to minimize the moisture content effect and to conform with the surface requirements of the HFM unit.

Additionally, to obtain the results of volumetric specific heat *c_v_* and thermal diffusivity *a*, tests were also performed using a transient method (C-Therm device, C-Therm Technologies Ltd. 40 Crowther Lane–Suite 200, Fredericton, New Brunswick, Canada E3C 0J1 https://ctherm.com/contact/). For this purpose, prism specimens with dimensions of 40 × 40 × 160 mm were produced. For each specimen type, a minimum of three slices, each 5 mm thick, were cut and placed in a dryer at a temperature of 70 °C until a constant weight was reached. The thermal parameters of the mortars were subsequently analyzed in a dry state. A surface probe was utilized for these measurements, which involved analyzing temperature fluctuations in the tested material through the flow of heat pulses.

The surfaces of the specimens designated for analysis were examined for flatness and parallelism to ensure proper adhesion of the measuring probe. For each sample surface, a minimum of three measurements were conducted to evaluate the repeatability of the obtained results. In addition to calculating the average values, standard deviations and coefficients of variation were also determined.

### 2.5. Compressive and Flexural Strength and Dynamic Elasticity Modulus

The bending test for each mixture was conducted on three prism-shaped specimens measuring 40 × 40 × 160 mm, in accordance with EN 1015-11 [40]. The final result of the test was the average of three measurements performed under controlled laboratory conditions. During the test, the prepared prisms were fractured into two sections. The resulting halves were subsequently utilized in repeated tests to ascertain the compressive strength of the mortar. The flexural strength was evaluated consecutively after 4, 28, and 90 days following the preparation of the specimens.

The compressive strength of the mortar was assessed in accordance with PN-EN 196-1 [41]. The standard specified a uniform load increase at a rate of 50 to 500 N/s, which was to be selected so that material failure occurred within a timeframe of 30 to 90 s. The arithmetic mean of all tested specimens (in this case, six) was considered reliable. Additionally, the determination of compressive strength was performed consecutively at 4, 28, and 90 days following the preparation of the specimens.

Finally, the dynamic modulus of elasticity of the mortars was defined following EN 12504-4 [42]. More precisely, the specimens’ ultrasonic pulse velocity was determined using the Proceq UPV instrument (Pundit Lab +, Screening Eagle Technologies AG Ringstrasse 2 8603 Schwerzenbach–Zurich, Switzerland, https://www.screeningeagle.com/en/contact-us). These non-destructive measurements were conducted at three prism specimens for each composition after 4, 28, and 90 hardening days.

### 2.6. Tests of the Water Absorption Coefficient Due to Capillary Rise

The test was conducted in accordance with the PN-EN 1015-18 standard using halves of rectangular specimens measuring 40 × 40 × 160 mm, which had silicone-insulated sidewalls. The specimens were positioned with the fractured surface submerged in a water tank. Weighing was performed at intervals of 10 and 90 min. The coefficient of water absorption due to capillary rise was calculated using the following formula [43]:

$$C=0.1\left(M2-M1\right)\mathrm{kg}/\left(m^{2}\cdot \min^{0.5}\right)$$

where *C*—water absorption coefficient [kg/m^2^·min^0.5^], *M*1—specimen’s weight after soaking for 10 min [g], and *M*2—specimen’s weight after soaking for 90 min [g].

### 2.7. Sorption and Desorption Tests

This test was conducted in accordance with the EN ISO 12571 standard [44]. The automated methods utilized for determining sorption isotherms serve as equivalents to traditional techniques [45]. Specifically, devices such as a Dynamic Vapor Sorption apparatus (DVS) (Surface Measurement Systems GmbH, Im Breitspiel 21, 69126 Heidelberg, Germany, https://surfacemeasurementsystems.com/contact/) were employed to generate moisture [46]. The apparatus monitored the change in mass over time (dm/dt) to ascertain the duration required to achieve equilibrium. When dm/dt approached zero, the system automatically adjusted to the subsequent humidity level. An air temperature of 20 °C and a relative humidity range of 0–97% were established, with humidity levels incremented by 10%. Each time the software detected a mass change of less than 0.0005% per minute, the relative humidity was automatically modified by 10%. Prior to testing, the specimens were dried to a constant weight at 70 °C, with an initial weight of approximately 150 µg. To ensure dry conditions at the commencement of the measurement, the test began with 12 h of sorption drying at a 0% humidity level within a nitrogen atmosphere.

### 2.8. SEM

A Hitachi TM3000 scanning electron microscope (SEM) (Hitachi High-Tech Europe GmbH, Europark Fichtenhain A 12, 47807 Krefeld, Germany) was utilized to evaluate the quality of the contact zone between the biowaste powders and the cement matrix in each composite. Specimens, measuring approximately 200 mm^2^ in area and up to 3 mm in thickness, were prepared for analysis. To enhance the electrical conductivity of the tested surfaces, the samples were coated with metal alloys in a vacuum chamber. Images were captured at magnifications ranging from 50× to 500×.

### 2.9. Mercury Porosimetry

Mercury intrusion porosimetry tests were conducted using the Quantachrome Poremaster 60 (Anton Paar Poland sp. z o.o.ul. Hołubcowa 123, Warszawa, 02-854, POLAND), following a curing period of 28 days for the composite materials. Specimens measuring 7 × 7 × 20 mm were extracted from the central section of larger prism specimens (40 × 40 × 160 mm). To ensure the reliability of the results, two tests were performed for each composite to assess repeatability. Prior to testing, the specimens were dried at 70 °C for 48 h. The mercury surface tension was set at 0.48 N/m, and the contact angle during intrusion was established at 140°. The specimens were placed into measurement cells and filled with mercury in a low-pressure chamber, which was maintained at pressures up to 0.34 MPa. Subsequently, the cells were transferred to a pressure chamber and subjected to high pressures reaching 413 MPa. The results yielded cumulative and log differential graphs illustrating the porosity distribution, as well as fundamental properties of the composites, including total porosity, specific surface area, and volume density.

### 2.10. Bacteria Growth

The Gram-negative bacterium *Escherichia coli*, strain K12 ATCC 25922, was utilized as the reference strain (American Type Culture Collection, ATCC). *E. coli* was cultivated in Enrichment Broth (BIOCORP Sp. z o.o., Poland, Skibicka 5, 02-269 Warszawa) and incubated at 37 °C for 24 h, followed by activation through two successive transfers. The overnight cultures of *E. coli* were then transferred to solutions prepared from reagent-grade chemicals (Chemland, Poland, UL. USŁUGOWA 3 73-110 STARGARD), specifically sterile saline buffer (0.85% NaCl). The culture was diluted with the appropriate buffer to achieve a final bacterial concentration ranging from approximately 1.5 to 3.0 × 10^6^ CFU/mL. All materials were evaluated in accordance with the standard test method for determining the antimicrobial activity of antimicrobial agents under dynamic contact conditions, with modifications based on ASTM E2149-20, “Standard Test Method for Determining the Antimicrobial Activity of Antimicrobial Agents under Dynamic Contact Conditions”, ASTM International: West Conshohocken, PA, USA, 2020.

Concrete plates 50 × 50 mm were sterilized under a UV-C lamp for 15 min. All specimens were placed into a sterile buffer in 250 mL screw-cap Erlenmeyer flasks. Then, 50 ± 0.5 mL of the working dilution of the prepared bacterial inoculum was added to the flask. The flask containing only bacterial solution was used as a negative control. The flasks on the wrist-action shaker at 37 °C were shaken at a maximum stroke for 3 h. An amount of 0.5 mL was taken as a sample after 0.5 (30 min), 1 (60 min), and 3 h (180 min). Bacterial concentration at the “0” time and during the experiment was measured by serial dilutions and standard plate-count techniques in triplicate. Plate Count Agar (BIOCORP Sp. z.o.o., PL, Skibicka 5, 02-269 Warszawa) was used. All Petri dishes were incubated at 37 ± 2 °C for 24 h. The visible bacteria colonies were counted and reported as colony-forming units per milliliter (CFU/mL). The logarithmic (log) bacteria reduction was calculated from the initial “0” time.

## 3. Experimental Results

### 3.1. SEM Results

**Figure 1:**
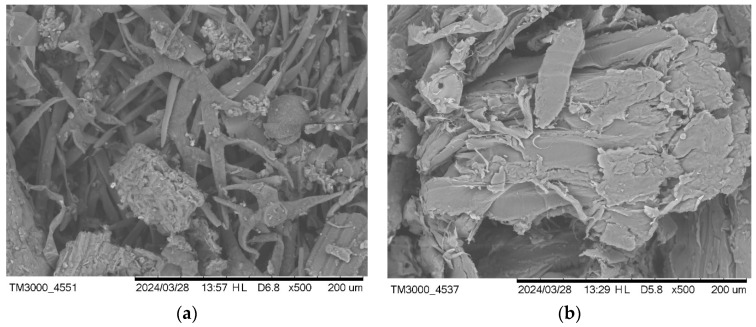
SEM images of lavender particles (**a**) and black pine particles (**b**).

The images from SEM (scanning electron microscope) displaying lavender particles (a) and black pine particles (b) are presented in Figure 1. The lavender particles exhibit a fibrous structure with varying dimensions, while the black pine particles appear to have a more layered, flaky texture. Both images were captured at a magnification of 500×, allowing a detailed examination of the particle morphology, which is essential for understanding how these biowaste additives interact with cement matrices.

### 3.2. Physical Properties (Density, Absorption, Open Porosity and Apparent Specific Gravity)

**Figure 2:**
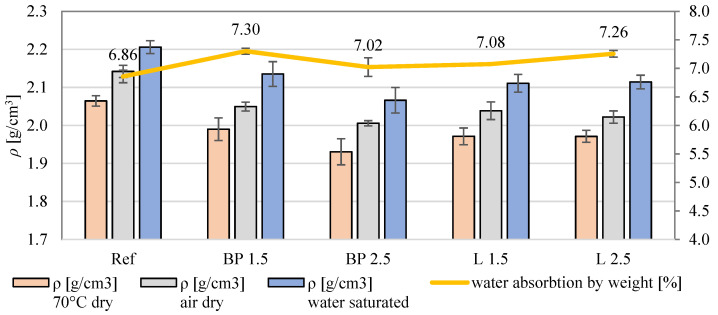
The average density and water absorbability of mortars with the addition of bio-additives.

In Figure 2, the average values of the densities of the tested mortars are presented. The dry density of all mortars with the addition of biowaste is lower than the density of the reference one. The most significant decreases in density were observed in mortars with biowastes BP 2.5 and L 2.5, which differ by approximately 4.5% compared to the reference mortar. The reduction in density is due to the lower mass of biowaste than the mineral aggregate used in the reference mortar.

The water absorption of all mortars with the addition of biowastes is higher than the water absorption of the reference mortar. The difference is the largest for mortars BP 1.5 and L 2.5, which are approximately 6% higher than in the REF. Increased water absorption may be caused by greater porosity of mortars with the addition of biowaste. This porosity may result from the breakdown of biowaste during the mixing and hydration process of the mortar and the formation of free spaces in the contact zone between the cement matrix and the additive. The results presented in the graph are consistent with the results of other studies on biomass’s impact on mortars’ properties. For example, the study [16] found that adding 5% biomass to cement mortar reduces its density by about 5% and increases water absorption by about 10%.

The porosity recorded (Table 3) was lower in the modified samples than in the REF samples, which indicates the cohesion achieved among the bio-powders and the matrix. The fine-grained bio-materials inside the cement matrix act as fillers [47] and contribute to decreasing porosity and absorption values. The highest porosity reduction was presented in cases BP 2.5 and L 1.5 at 28 and 90 days (about −39% and −54%, respectively). Regarding the specific gravity of the specimens, the implementation of bio-powders in the mortar mixture induced lightness in the final samples. The addition of BP powder led to a denser structure in relation to L powder at 90 days. Additionally, the increase in the content of bio-materials seems to improve the density of the structure further, especially in the samples with black pine powder (about a 2% reduction at the BP 2.5 case compared to BP 1.5 at the age of 90 days).

### 3.3. Thermal Properties

The bio-additives also influenced the thermal properties of the tested mortars. The results of thermal conductivity are shown in Table 4. The results from the heat flux meter are presented for specimens tested after 28 and 90 days. In general, the addition of powders resulted in a decrease in thermal conductivity after 28 days of curing. The biggest drop can be observed in specimens with black pine powder, where λ decreased by 22% compared to the reference mortar.

After 90 days of curing and conditioning the specimens in laboratory air-dry conditions, the differences in thermal conductivity are smaller. This is because the composites with the bio-powders have higher sorption properties, thus absorbing more water vapor from the air. As a result, its thermal conductivity was higher than after 28 days.

**Figure 3:**
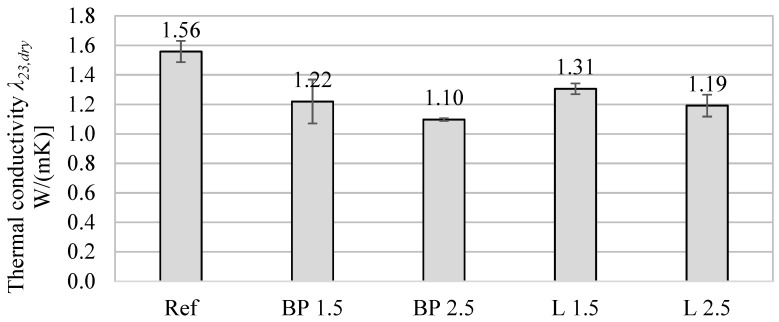
The average thermal conductivity of mortars containing bio-additives.

**Figure 4:**
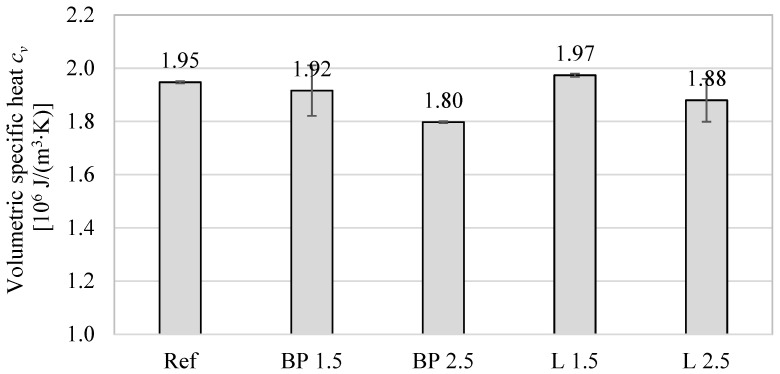
The average volumetric specific heat of mortars containing bio-additives.

**Figure 5:**
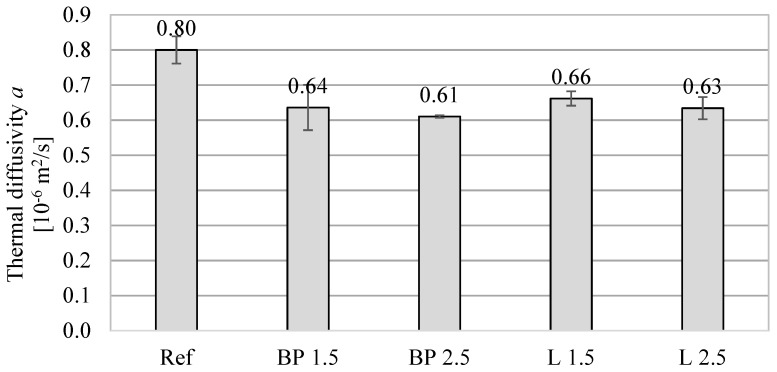
The average thermal diffusivity of mortars containing bio-additives.

The thermal properties results obtained using the transient method are shown in Figure 3, Figure 4 and Figure 5. Due to a different testing methodology, the results differ from those of the heat flux meter. However, there is still a significant tendency for the thermal conductivity to decrease with the increase in bio-additive content. The lowest values were obtained with 2.5% bio-additives content. The reduction in conductivity was 30% in the mortar with the addition of black pine and 23% in the mortar with lavender waste, respectively. The results are confirmed by data included, among others, in the paper [24], where the relatively low thermal conductivity of biowaste, including lavender waste, was indicated.

The influence of bio-additives on the volumetric specific heat of powder-containing composites is worth noticing. Despite the greater porosity of mortars with biowaste, in the case of 1.5% addition, their effect is rather positive, and they even cause a slight increase in specific heat (L 1.5). A more considerable amount of powder causes a 4–7% decrease in specific heat compared to the reference mortar without additives. Therefore, using mortars with biowaste as an interior finishing material should not negatively impact reducing the internal heat capacity of the walls and other partitions inside the building.

In turn, the thermal diffusivity of all mortars with the addition of bio-powders is lower by approximately 17–23%, depending on bio-additive content. This is mainly caused by the lower thermal conductivity of composites with biowaste.

### 3.4. Microstructural Properties

**Figure 6:**
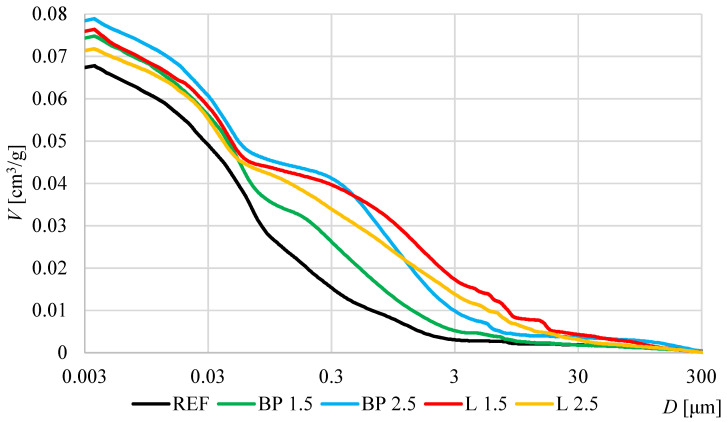
Cumulative porosity graphs of the tested mortars with biowastes (MIP).

**Figure 7:**
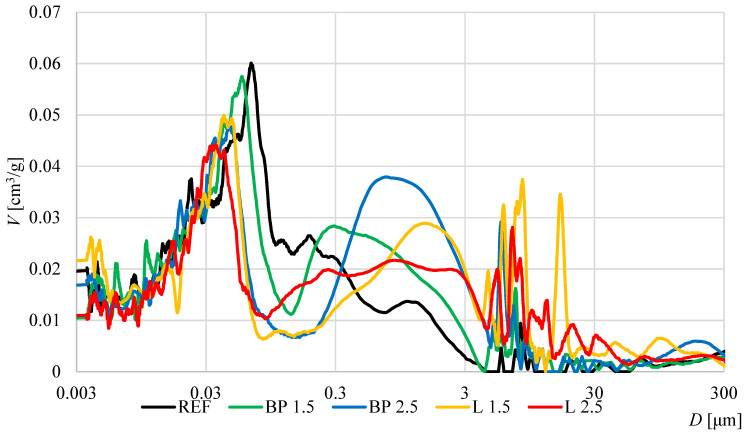
Log-differential porosity graphs of the tested mortars with biowastes (MIP).

The porosimetry results are presented in Figure 6 and Figure 7 and Table 5. In all composites with the addition of bio-powders, a significant increase in pore content was observed compared to the reference mortar. In the case of black pine powder, a correlation is visible between the increase in porosity and the additive content.

However, no such correlation was obtained concerning the results of composites with lavender powder. The pore content is higher in both composites (L 1.5 and L 2.5) than in the REF mortar. Due to the addition of a huge amount of superplasticizer in the L 2.5 mortar, which is necessary to obtain the appropriate consistency of the fresh mixture, its porosity turned out to be lower than in the analogous L 1.5 mortar.

Two ranges of significantly increased pore content of mortars with bio-powders are visible. The first one concerns the range between 3 and 30 μm and reflects the entrained air pore created by mixing the powder with a fresh mortar mixture—large pores were formed at the interface between the powder and the cement matrix. The second interval is observed in the range between 0.3 and 3 μm and represents the biowaste powders’ porosity.

It should also be noted that bio-powders also caused a decrease in porosity in the range from 30 to 70 nm. Compared to the reference mortar, the extreme in this range is lower and with a shift towards tiny capillary pores (from 70 nm in the REF to 30 nm in L 2.5). This affects the hygroscopic properties of individual mortars, which is discussed later in the text.

The addition of bio-powders also influenced other microstructure properties of the composites. With the increase in powder addition, the total specific surface area decreased significantly—which is caused by, among others, lower porosity in the range of 30–70 nm. A significant change in pore permeability was also observed. Using powders resulted in the formation of connections between individual pores and a general increase in porosity, which increased pore permeability.

### 3.5. Mechanical Properties

**Figure 8:**
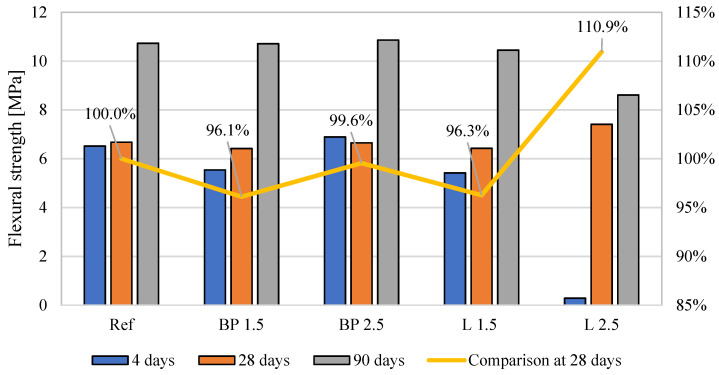
The average flexural strength of mortars containing biowastes.

**Figure 9:**
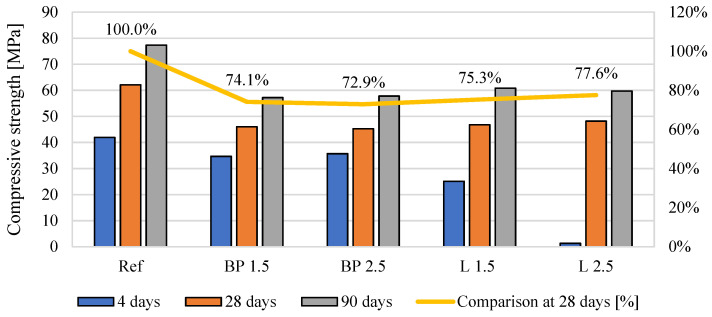
The average compressive strength of mortars containing biowastes.

**Figure 10:**
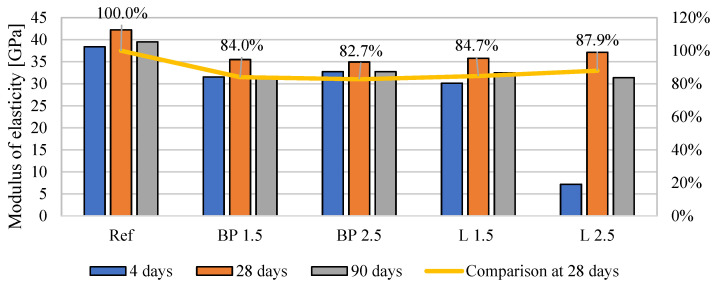
The average flexural strength of mortars containing biowastes.

The results of the mechanical properties are shown in Figure 8, Figure 9 and Figure 10. In the case of flexural strength, the addition of biowaste does not have a significant impact on the results. A slight 4% decrease in strength is observed with a 1.5% bio-powder addition after 28 days.

After 90 days of curing, the results are almost identical to the reference mortar. The exception here is the L 2.5 mortar, where after 28 days of curing, there is a 10% increase in strength. However, a decrease in strength after 90 days can be observed in that composite, which may indicate a certain breakdown of powders over a more extended period and, thus, worse strength results.

In the case of compressive strength, a clear decrease in strength is visible, from 22 to 27%, after 28 days of curing. There are no high differences here between 1.5 and 2.5% of biowaste. The result for 2.5% lavender content is even better than other composites with biowaste. It should be taken into account that L 2.5 used much larger amounts of superplasticizer and, therefore, changed the porosity structure of the cement matrix and thus mitigated the effect of the bio-additive itself.

The negative effect of the superplasticizer in L 2.5 is visible in the results after 4 days of curing. Too much of the additive caused a problem with cement hydration, which was visible in the results of this mixture compared to the properties of the other composites. However, after 28 days, this effect disappears, and the L 2.5 mixture does not differ significantly from the others. In the case of the elastic modulus, all mortars with bio-additives were characterized by a 16–18% lower modulus than the reference mortar. A similar trend is maintained in the results from 90 days of curing.

### 3.6. Antibacterial Properties

**Figure 11:**
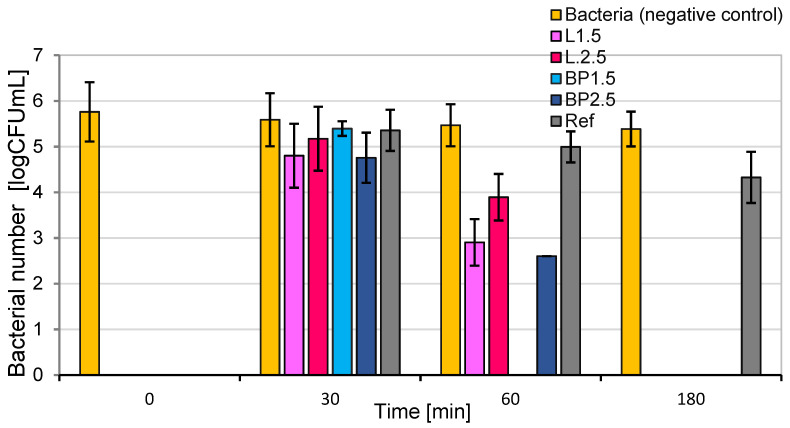
Survival rate of the mortars containing varied amounts of lavender (L) and black pine powder (BP); references sample (REF) towards *Escherichia coli*.

The antibacterial properties against *E. coli* are presented in Figure 11.

The mortars containing 1.5 and 2.5% lavender powder (L) and black pine (BP) and those without additives were tested for their antibacterial performance via the dynamic contact conditions method. All the mortars (except the negative control and references sample) presented excellent antibacterial properties against model Gram-negative bacteria *Escherichia coli* after 180 min. The most effective method for *E. coli* was mortar containing 1.5% of pine needles (BP). After one h (60 min), the bacterial number reduction was higher than 5 logCFU/mL. The presence and size of pores are the second most important factors that enhance bacterial growth (Table 5). Otherwise, a fast decrease in bacterial number observed for 1.5 BP mortar may be due to a more accessible contact between bacteria and aromatic pine wastes inside the large pores. The sample containing 1.5% pine needles was characterized by the highest content (7.93%) of millipores (100 μm–100 mm) in comparison to other tested materials containing lavender and pine powders (Table 5).

On the other hand, it may be due to high water absorption by weight (7.30%), leading to the absorption of some bacteria suspension where the sample was immersed (Figure 2). A similar relationship was found for mortars containing lavender powder. However, the higher water absorption by weight of mortar containing 2.5% of lavender powder, in this case, was caused by a high content of micropores (100 nm–100 μm) (Figure 2, Table 5). It was concluded that a combination of two effects, such as plant waste content and water absorption (depending on porosity), is responsible for the antibacterial properties of obtained materials.

The antibacterial properties of mortars containing inorganic nano-additives, e.g., TiO_2_, ZnO, CuO, SiO_2,_ or other metal compounds (silver molybdate, copper oxide, zinc oxide, sodium tungstate, or sodium bromide) are described in the literature quite frequently. Antimicrobial building materials, with the addition of diverse organic antimicrobial agents, less attention is paid [48]. Also, there is a knowledge gap regarding incorporating lavender and pine powders into concrete to increase its resistance to biodeterioration. However, the antimicrobial activity of lavender and pine essential oils incorporated into polymer structures is well known, and it has been the focus of several studies [49,50,51]. It is well known that pine and lavender powders are rich in essential oils and can be used as a good source of naturally occurring secondary metabolites with antibacterial properties [49,50,52]. It was proved that the main antibacterial agents are monoterpenoids, such as thymol, carvacrol, α-pinene, β-pinene, or terpene alcohols, e.g., geraniol, alpha-terpineol, linalool, or esters, e.g., bornyl acetate and linalyl acetate [49,53].

The results obtained in this study confirm that adding aromatic plants’ wastes to cement is a viable method of inhibiting the growth of microorganisms. According to Adebanjo et al., formulating efficient antimicrobial agents (AMAs) is cost-effective in comparison to inorganic AMAs, does not cause any health concerns among end-users (is less hazardous), and does not require any regulatory frameworks [54].

### 3.7. Moisture-Related Properties

**Figure 12:**
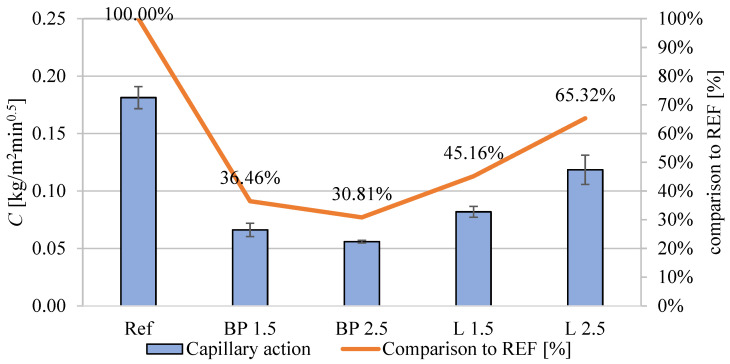
Capillary action of mortars containing biowastes.

Figure 12 shows a comparison of capillary action properties of mortars with the addition of biowaste. Unlike water absorption, capillary absorption is much lower in mortars with the addition of bio-powders. The impact of biowaste is evident in composites with black pine particles of a volume fraction of 2.5%; capillary action in the BP 2.5 composite is only 31% of the value of the reference mortar. The effect is most likely caused by certain hydrophobic properties of the oils contained in the biowaste powders. It may also be caused by a lower content of capillary pores in the range of 30–80 nm.

A much smaller decrease than in the REF was observed in L 2.5, which is most likely caused by using a large amount of superplasticizer; the fluidizing admixture makes this mortar behave entirely differently than its L 1.5 equivalent.

**Figure 13:**
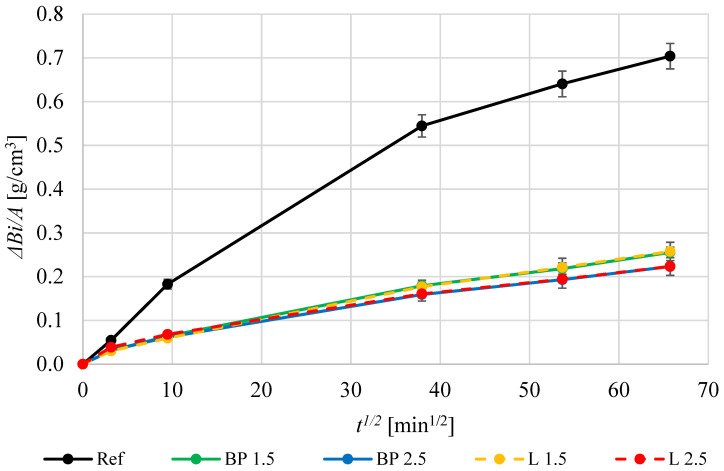
Capillary absorption curves of the produced mortars at 28 days.

Capillary action results from over 72 h were also compared (Figure 13). There is a significant difference between the reference mortar and those with the addition of bio-powders. The use of lavender or black pine powder results in a significant reduction in capillary wicking compared to the REF mortar. The differences between the types of bio-powders and their volume (i.e., 1.5% or 2.5%) are insignificant. Even 1.5% of the powder volume results in a significant reduction in wicking both for lavender and black pine. After 72 h of tests, the differences in the weight change in the specimens exceed 180% of the value in relation to the reference mortar. Therefore, bio-powders tend to block capillary action.

**Figure 14:**
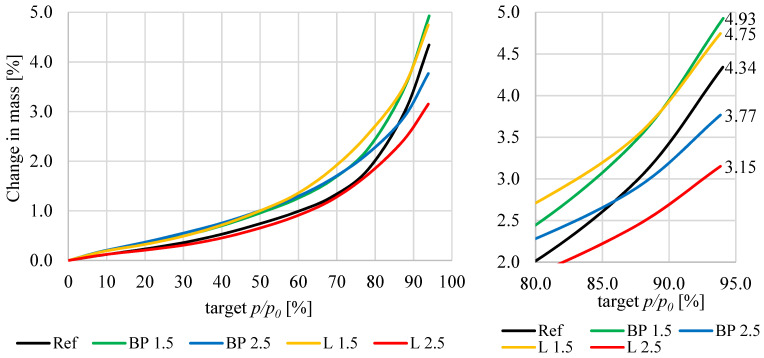
Sorption isotherms of the tested mortars.

**Figure 15:**
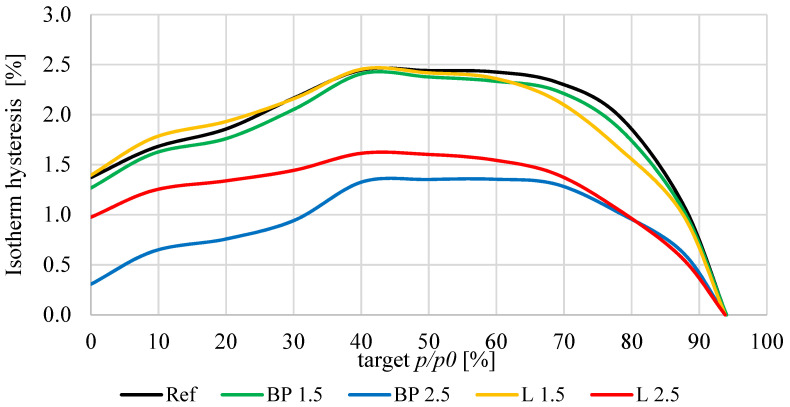
Hysteresis curves for the tested mortars.

The sorption properties of mortars with bio-powders look different than capillary action. The sorption diagram for the humidity ranges from 0 to 95%, and the hysteresis curves are shown in Figure 14 and Figure 15. The factor that significantly influenced the obtained sorption properties was the specific surface area of individual mortars. In the case of bio-powder additions at the level of 1.5% of the mortar volume, increased porosity in the range of nanopores and micropores and a slightly higher specific surface area resulted in a significant increase in sorption moisture in the whole range of air humidity in comparison to the REF.

However, in the case of adding 2.5% of the biowaste volume, their content decreased the total specific surface area, which consequently reduced the sorption moisture of these composites—especially in terms of high relative air humidity.

Mortars containing bio-powders at 1.5% by volume behave similarly to the reference mortar without biowaste. Adding 2.5% causes a significant difference in the hysteresis curve (sorption-desorption). These mortars do not accumulate as much moisture in their volume as the REF, and during the drying process, they also release more moisture into the environment.

## 4. Discussion

**Figure 16:**
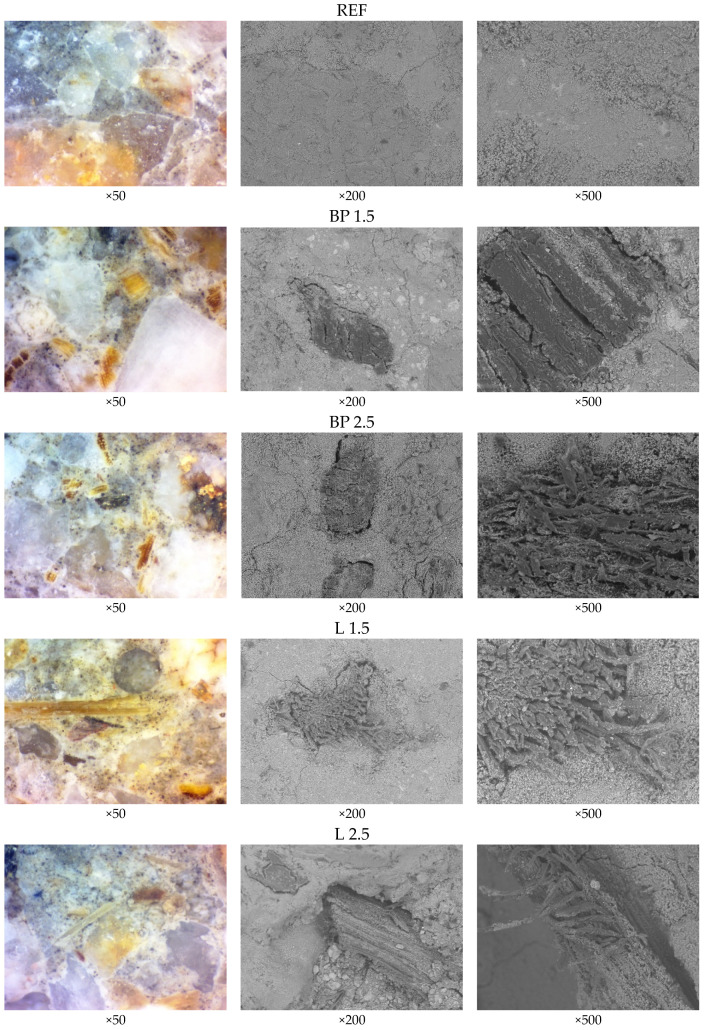
Structure of tested mortars under stereoscopic microscope observation (50× zoom) and SEM observation (200× and 500× zoom).

In Figure 16, the cross-sections of each tested mortar type are compared using an optic stereoscope and scanning electron microscope. In the specimen of the REF mortar, the surface is smooth and homogeneous. There are no pores or cracks visible in the mortar structure. The aggregate grains are evenly distributed and well-bonded with the cement matrix. When comparing the cross-sections of individual types of mortars, differences between the addition of lavender and black pine waste are visible. In the case of a 1.5% content of both black pine and lavender, minor separations are observed between the biowaste and the cement matrix. This phenomenon explains the reduced mechanical strength and increased porosity of these mortars. In contrast, in the images of mortars with a 2.5% lavender content, significant detachments are visible between the matrix and the biowaste. As a result, there is a marked increase in capillary absorption, as demonstrated in the following graphs.

Images from the stereoscope show small and quite regular particles of black pine. However, in the case of lavender waste, the powders are much longer and cut along the cement matrix. In the case of black pine powder, the individual particles are relatively evenly distributed throughout the cross-section of the analyzed specimens. However, in the mortar containing lavender, the fibers, due to their length, tend to “cut” through the cement matrix, resulting in a less uniform distribution across the cross-section.

In the case of photos taken using SEM, a better bond between the contact layer of the cement matrix and the inserts in BP composites is also visible. On the contrary, in the L 2.5 mortar, large gaps between the cement matrix and the fibers are visible, which negatively affects the mechanical properties.

**Figure 17:**
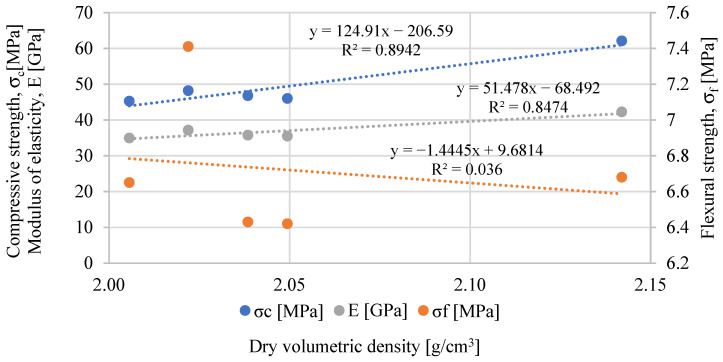
Relationship between volumetric density and mechanical properties.

When comparing the effect of density on mechanical properties, the expected tendency was observed in which, with the decrease in density caused by the use of bio-powders, the compressive strength after 28 days of curing and the modulus of elasticity was lower than in the reference mortar (Figure 17). However, such a relationship was not observed in the case of flexural strength. After adding the powders, their effect on strength was neutral.

**Figure 18:**
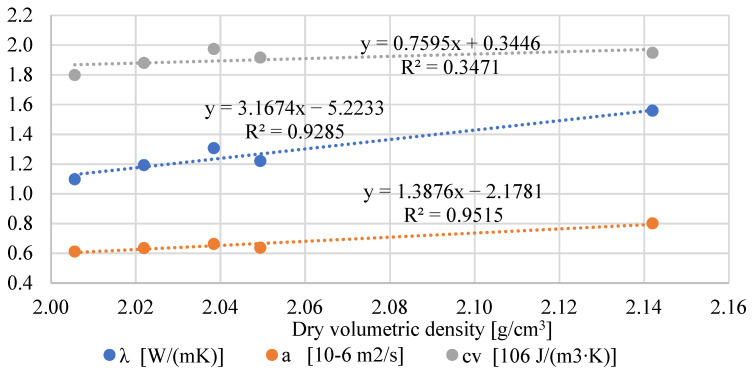
Relationship between volumetric density and thermal properties.

In the case of thermal properties (Figure 18), a strong relationship is visible between thermal conductivity, thermal diffusivity, and volumetric density. Lowering the density (and simultaneously increasing the material’s porosity) resulted in a decrease in thermal conductivity. However, the influence of adding powders on the volumetric specific heat is not apparent. On the one hand, the decrease in density causes a decrease in specific heat. On the other hand, lavender and black pine powders are characterized by a much higher specific heat than the cement matrix. This, in turn, results in an even higher specific heat (L 1.5) than in the reference mortar.

**Figure 19:**
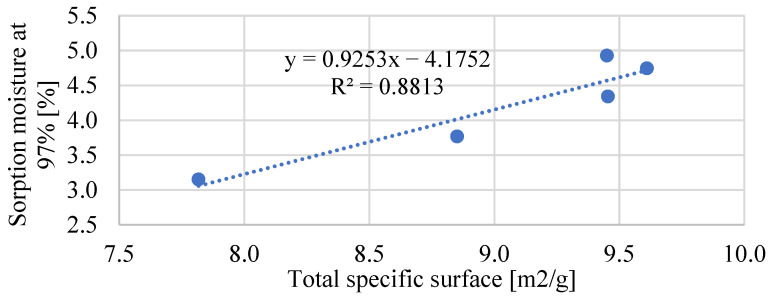
Relationship between total specific surface and sorption moisture obtained at 97% of air humidity.

A solid relationship between the total surface area of mortars and their sorption properties was also noticed. Figure 19 shows the relationship between the specific surface and the sorption moisture obtained at an air humidity of 97%. In the case of mortars in which the powder content was 2.5%, the specific pore surface was significantly lower, which resulted in lower sorption capacity of these materials. Therefore, this type of bio-powder may be justified wherever the air humidity is relatively high, and the materials should be characterized by low sorption—increasing their resistance to moisture.

Table 6 presents the rough estimation of CO_2_ emissions of individual raw materials for mortar production. It was assumed that OPC emits 780 kg/tone and river sand 5.5 kg/tone of CO_2_ [55,56,57]. As confirmed by EDS analysis, the powders used consist of 96% C and O. Therefore, it was assumed that adding powders to the mortar would store CO_2_ in an amount corresponding to the weight of the biowaste used, thus reducing the total CO_2_ emission of mortars. This simple analysis indicates an additional benefit of using powder in the form of a 4.2 and 7.1% reduction in the CO_2_ emission of the mortar with the addition of biowaste, respectively.

## 5. Conclusions

Based on the obtained results and discussion, the following conclusions may be formulated:

1. The incorporation of biowaste results in a reduction in thermal conductivity by approximately 30% when black pine powder is used and up to 23% with the addition of lavender powder.
2. The inclusion of bio-powders at 1.5% of the mortar volume does not significantly affect the volumetric specific heat; however, higher concentrations result in a decrease in specific heat by 4–7%.
3. The use of bio-powders led to an increase in porosity within the range of 3 nm to 300 μm by 0.5–1.5%, while simultaneously causing a significant reduction in the total specific surface area of the composites and enhancing pore permeability.
4. An increase was observed in the capillary pore range (100 nm to 100 μm), while larger pores (>100 μm) showed a decrease with the incorporation of biofibers.
5. The addition of bio-powders did not result in a notable impact on flexural strength after 28 days of curing, but compressive strength decreased significantly by 22–27% after the same curing period.
6. Antimicrobial properties were enhanced by the addition of 1.5% to 2.5% lavender or black pine powder.
7. Mortar samples containing 1.5% pine powder demonstrated superior antimicrobial properties compared to other tested samples.
8. In composites containing 1.5% and 2.5% bio-powder by volume, a significant reduction in capillary action was observed in mortars exposed to water for both short-term (90 min) and extended periods (three days).
9. Higher bio-powder content (2.5%) also resulted in a reduction in sorption properties at elevated levels of air humidity.
10. The use of waste bio-powders contributes to a reduction in the mortar’s CO_2_ emissions by 4–7%.

The incorporation of bio-powders, such as black pine powder and lavender, offers a sustainable approach to managing waste from the cosmetics industry, significantly reducing the volume of waste generated. The addition of these powders enhances certain properties of traditional mortars, including reduced capillary action, lower sorption rates, improved bactericidal properties, and maintained flexural strength. Furthermore, embedding these waste materials within the mortar structure contributes to a reduction in CO_2_ emissions associated with the production of ordinary Portland cement, providing an environmentally beneficial alternative.

## Figures and Tables

**Table 1:** Mix compositions (mass proportions).

Composition Name	CEM I 42.5 R	River Sand (0–4 mm)	Bio-Material (% *v*/*v*)	w/c	Superplasticizer (% *w*/*w*)	Workability (cm)	Consistency Class
REF	1	2.5	-	0.45	-	12.5	F1
BP 1.5	1	2.5	black pine powder 1.5%	0.45	0.6	13.0	F1
BP 2.5	1	2.5	black pine powder 2.5%	0.45	1.5	11.5	F1
L 1.5	1	2.5	lavender powder 1.5%	0.45	0.7	12.5	F1
L 2.5	1	2.5	lavender powder 2.5%	0.45	3.0	10.5	F1

**Table 2:** Elemental composition of the biowastes.

Material	Cr [wt. %]	Na [wt. %]	Cd [wt. %]	K [wt. %]	Zn [wt. %]	Cu [wt. %]	Mg [wt. %]	Mo [wt. %]	Mn [wt. %]	Ca [wt. %]
Lavender powder	0.0001	0.0047	-	0.9593	0.0021	0.0008	0.3718	0.0000	0.0028	1.2057
Black pine powder	0.0002	0.0008	0.0000	0.0126	-	0.0003	0.0089	0.0001	0.0007	0.0892

**Table 3:** Physical properties of the produced mortars at 28 and 90 days.

Specimen	Age [Days]	Absorption (%)	Open Porosity (%)	Specific Gravity [-]
REF	28	2.96	6.42	2.17
BP 1.5		1.98	4.09	2.06
BP 2.5		1.87	3.90	2.09
L 1.5		2.04	4.31	2.12
L 2.5		2.07	4.37	2.12
REF	90	3.05	6.83	2.24
BP 1.5		2.13	4.49	2.11
BP 2.5		2.04	4.21	2.07
L 1.5		2.87	3.13	2.09
L 2.5		2.58	3.40	2.05

**Table 4:** Thermal conductivity coefficient (λ) of the produced mortars at 28 and 90 days, at 10 °C and 20 °C (mean heating temperature).

Specimen	Age [Days]	λ [W/(mK)]	
		10 °C	20 °C
REF	28	1.2354	1.2644
BP 1.5		0.9549	0.9770
BP 2.5		0.9489	0.9827
L 1.5		1.1946	1.2183
L 2.5		1.1435	1.1651
REF	90	1.2333	1.2147
BP 1.5		1.1222	1.0819
BP 2.5		1.0750	1.0690
L 1.5		1.2264	1.2426
L 2.5		1.1746	1.1203

**Table 5:** Porosity properties derived from mercury porosimetry analysis and volume densities.

Type	REF	BP 1.5	BP 2.5	L 1.5	L 2.5
Total specific surface [m^2^/g]	9.454	9.450	8.851	9.610	7.817
Pore tortuosity [-]	2.09	2.05	2.019	2.0024	1.9773
Permeability [10^−4^ nm^2^]	0.0003	0.0005	0.0008	0.0007	0.0009
Porosity (3 nm–300 μm) [%]	14.46	14.97	15.90	15.40	15.04
Nanopore (0.1 nm–100 nm) [%]	0.28	0.24	0.56	0.40	0.29
Micropore (100 nm–100 μm) [%]	5.44	6.81	8.50	8.39	8.48
Millipore (100 μm–100 mm) [%]	8.74	7.93	6.83	6.61	6.26
Volume density, mercury porosimetry [g/cm^3^]	2.13	2.00	2.02	2.02	2.09

**Table 6:** CO_2_ emissions per 1 m^3^ of mortar.

No.	Name	Cement [kg CO_2_]	Sand [kg CO_2_]	Bio-Powders [kg CO_2_]	Total Emission [kg CO_2_]	Comparison to REF [%]
1	REF	454.6	8.0	0.0	462.6	0.0
2	BP 1.5	454.6	8.0	−19.6	443.0	−4.2
3	BP 2.5	454.6	8.0	−32.6	429.9	−7.1
4	L 1.5	454.6	8.0	−19.6	443.0	−4.2
5	L 2.5	454.6	8.0	−32.6	429.9	−7.1

## Data Availability

The original contributions presented in the study are included in the article, further inquiries can be directed to the corresponding author.
